# Evolution of cis-regulatory modules for the head organizer gene *goosecoid* in chordates: comparisons between *Branchiostoma* and *Xenopus*

**DOI:** 10.1186/s40851-019-0143-1

**Published:** 2019-08-02

**Authors:** Yuuri Yasuoka, Yukiko Tando, Kaoru Kubokawa, Masanori Taira

**Affiliations:** 1 Department of Biological Sciences, Graduate School of Science, University of Tokyo, 7-3-1 Hongo, Bunkyo-ku, Tokyo, 113-0033 Japan; 2 Marine Genomics Unit, Okinawa Institute of Science and Technology Graduate University, 1919-1 Tancha, Onna-son, Okinawa, 904-0495 Japan; 3 Laboratory for Comprehensive Genomic Analysis, RIKEN Center for Integrative Medical Sciences, 1-7-22 Suehiro-cho, Tsurumi-ku, Yokohama, 230-0045 Japan; 4 Center for Advance Marine Research, Ocean Research Institute, The University of Tokyo, 1-15-1, Minamidai, Nakano-ku, Tokyo, 164-8639 Japan; 5 Present address: Cell Resource Center for Biomedical Research, Institute of Development, Aging and Cancer (IDAC), Tohoku University, 4-1 Seiryo-machi, Aoba-ku, Sendai, Miyagi 980-8575 Japan; 6 Present address: SIRC, Teikyo University, 2-11-1, Itabashi-ku, Tokyo, 173-8605 Japan; 7 Present address: Department of Biological Sciences, Faculty of Science and Engineering, Chuo University, 1-13-27 Kasuga, Bunkyo-ku, Tokyo, 112-8551 Japan

**Keywords:** Spemann-Mangold organizer, Gene regulatory network, Genomics, Vertebrate, Cephalochordate, Nodal/FoxH1 signaling, Lim1, Otx, Chordin

## Abstract

**Background:**

In cephalochordates (amphioxus), the notochord runs along the dorsal to the anterior tip of the body. In contrast, the vertebrate head is formed anterior to the notochord, as a result of head organizer formation in anterior mesoderm during early development. A key gene for the vertebrate head organizer, *goosecoid (gsc)*, is broadly expressed in the dorsal mesoderm of amphioxus gastrula. Amphioxus *gsc* expression subsequently becomes restricted to the posterior notochord from the early neurula. This has prompted the hypothesis that a change in expression patterns of *gsc* led to development of the vertebrate head during chordate evolution. However, molecular mechanisms of head organizer evolution involving *gsc* have never been elucidated.

**Results:**

To address this question, we compared cis-regulatory modules of vertebrate organizer genes between amphioxus, *Branchiostoma japonicum*, and frogs, *Xenopus laevis* and *Xenopus tropicalis*. Here we show conservation and diversification of gene regulatory mechanisms through cis-regulatory modules for *gsc*, *lim1/lhx1*, and *chordin* in *Branchiostoma* and *Xenopus*. Reporter analysis using *Xenopus* embryos demonstrates that activation of *gsc* by Nodal/FoxH1 signal through the 5′ upstream region, that of *lim1* by Nodal/FoxH1 signal through the first intron, and that of *chordin* by Lim1 through the second intron, are conserved between amphioxus and *Xenopus*. However, activation of *gsc* by Lim1 and Otx through the 5′ upstream region in *Xenopus* are not conserved in amphioxus. Furthermore, the 5′ region of amphioxus *gsc* recapitulated the amphioxus-like posterior mesoderm expression of the reporter gene in transgenic *Xenopus* embryos.

**Conclusions:**

On the basis of this study, we propose a model, in which the *gsc* gene acquired the cis-regulatory module bound with Lim1 and Otx at its 5′ upstream region to be activated persistently in anterior mesoderm, in the vertebrate lineage. Because Gsc globally represses trunk (notochord) genes in the vertebrate head organizer, this cooption of *gsc* in vertebrates appears to have resulted in inhibition of trunk genes and acquisition of the head organizer and its derivative prechordal plate.

**Electronic supplementary material:**

The online version of this article (10.1186/s40851-019-0143-1) contains supplementary material, which is available to authorized users.

## Background

The Phylum Chordata, named for the presence of the notochord on the dorsal side during embryogenesis, consists of three subphyla: Cephalochordata, Urochordata, and Vertebrata. Cephalochordates (amphioxus) are so named because the notochord extends to the anterior tip of the body (“cephalo-” denotes “head”). In the Vertebrata, the head is formed anterior to the notochord, suggesting evolutionary development of a “new head” in the space anterior to the notochord [1, 2]. This head includes two lobes of the telencephalon, paired eyes, placodes, and cranial neural crest cells, which are lacking in amphioxus [3, 4]. Urochordates (tunicates) are quite diverse in morphology, despite their phylogenetic position as a vertebrate sister group. Therefore, amphioxus is a basal invertebrate chordate and the ancestral vertebrate was thought to be amphioxus-like [3, 4]. Thus, comparisons of developmental systems between amphioxus and vertebrates have provided important insights into the evolution of the vertebrate head. Although the definition of the vertebrate head remains problematic and involves many anatomical features, such as nerves, skeletal elements, and muscles, here we simply characterize the vertebrate head as the anteriorly enlarged central nervous system (forebrain) derived from anterior neuroectoderm, which is formed during gastrulation.

To date, based on comparative analyses of gene expression patterns and neuroanatomy, the rostral part of amphioxus central nervous system is postulated to be homologous to that of vertebrates, suggesting that the vertebrate brain was acquired on a foundation already present in the ancestral chordate, instead of through addition of a new part anterior to the notochord [5]. Even in hemichordates, a non-chordate deuterostome lineage, gene expression patterns along the anteroposterior (AP) axis in ectoderm are similar to those in vertebrate central nervous system, suggesting a deep ancestry of the molecular networks underlying AP axis formation in deuterostome nervous systems [6, 7]. In addition, abundant molecular evidence suggests that the lateral plate ectoderm of tunicates shares evolutionary origin with vertebrate neural crest and cranial placodes [8]. These recent molecular data argue strongly against the “new head” theory, which asserts that neural crest and neurogenic placodes are unique to vertebrates, and that most of the vertebrate forebrain are neomorphic structures [1, 2]. Therefore, we need to investigate how the head region was converted from the amphioxus-type to the vertebrate-type. To address this question, we focused on head organizer genes in vertebrates, *otx2, lim1* (also called *lhx1*), *goosecoid* (*gsc*), and *chordin* (*chrd*), by comparing their gene regulatory networks (GRNs) during head formation in frogs (*Xenopus*) and amphioxus.

In vertebrate embryogenesis, the head region is induced during the gastrula stage by the head organizer, which is the anterior part of the gastrula organizer (the Spemann-Mangold organizer in amphibians, the shield in teleosts, and the mid-gastrula organizer in mice). It should be noted that, in mouse embryos, anterior visceral endoderm is also involved in the anterior patterning despite the absence of axis inducing activity [9]. To understand head evolution in chordates, we focused on molecular mechanisms underlying head organizer formation, following gastrula organizer formation. In *Xenopus* and zebrafish, the gastrula organizer is formed in the late blastula stage by maternal canonical Wnt signaling from the dorsal region and Nodal signaling from the dorsovegetal region, which induce so-called organizer genes encoding transcription factors such as Otx2, Lim1, and Gsc, as well as Bmp antagonists such as Noggin, Chrd, and Cerberus involved in dorso-ventral (DV) patterning [10–13]. During gastrulation, the organizer is gradually divided into head and trunk organizers, which promote antero-posterior (AP) patterning of neuroectoderm [14, 15]. The head organizer induces forebrain and midbrain formation and also determines the anterior midline [16]. Head and trunk organizer regions develop into the prechordal plate and the notochord, respectively. The prechordal plate is a vertebrate-specific tissue that escapes from convergent extension movements occurring in notochordal cells.

The homeobox gene *gsc* is known as a head organizer-specific and later, a prechordal plate-specific gene, and is necessary for repressing trunk organizer genes and ventral genes such as *brachyury, wnt8,* and *ventx genes* [17–21]. The trunk organizer gene, *brachyury*, is a crucial gene for notochord formation [22]. Knockdown analysis of *gsc* in *Xenopus* embryos results in a short head and a cyclops phenotype, caused by reduction of the prechordal plate [20, 21]. A recent genome-wide study by our group using *Xenopus tropicalis* embryos also showed that Gsc binds to thousands of genomic regions and represses many trunk genes in concert with Otx2 and TLE/Groucho corepressor in the head organizer of early gastrula embryos [21]. Thus, Gsc is thought to be a key regulator of the head organizer and of prechordal plate development.

**Fig. 1:**
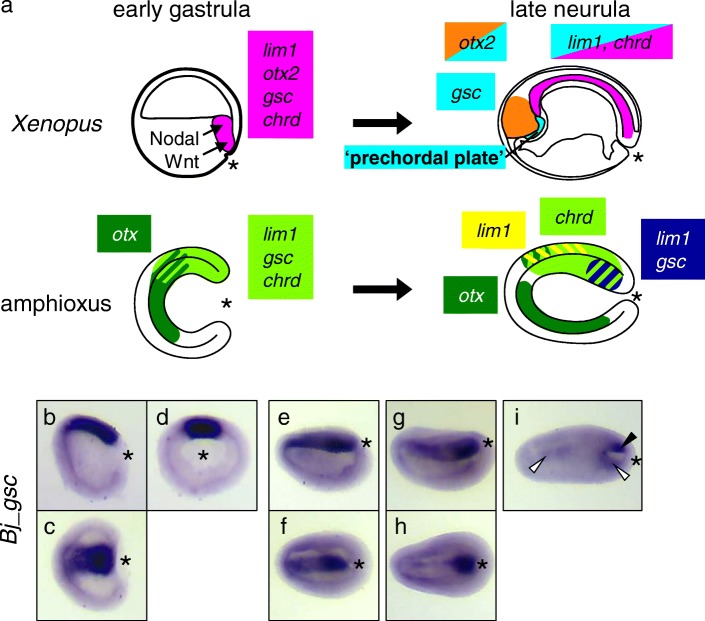
Schematic representations of organizer gene expression patterns. **a** Expression patterns of *lim1*, *otx2* (*otx in amphioxus*), *goosecoid* (*gsc*), and *chordin* (*chrd*) of *Xenopus* (top) and amphioxus (bottom) at the early gastrula stage (left) and the late neurula stage (right) are shown with colors as indicated. **b**–**i** Whole-mount in situ hybridization of *gsc* in *B. japonicum* embryos. In mid-gastrula (stage G5–6), *Bj_gsc* is expressed in the dorsal mesoderm (**b**–**d**). In late gastrula (stage G7–N0), *Bj_gsc* is still expressed throughout the dorsal mesoderm (**e**, **f**). In early neurula (stage N1), *Bj_gsc* expression is still strong in the posterior mesoderm but very weak in the anterior mesoderm (**g**, **h**). In mid-neurula (stage N2), *Bj_gsc* is expressed in the posterior mesoderm (arrowhead) and weakly in the dorsal endoderm (open arrowheads) (**i**). Embryos are shown in lateral view with dorsal to the top and anterior to the left (B, **e**, **g**, **i**), dorsal view with anterior to the left (**c**, **f**, **h**), or blastoporal view with dorsal to the top (**d**). *, blastopore

In amphioxus, recent extensive studies of expression patterns of many developmental regulatory genes and roles of cellular signaling during early embryogenesis have shown that fundamental mechanisms of DV and AP axis formation and expression patterns of organizer and non-organizer genes are highly conserved between amphioxus and vertebrates [23–26]. For example, *chrd* is expressed in dorsal mesoderm and ectoderm [24]. The LIM homeobox gene *lim1/5* (an ortholog of vertebrate paralogs *lim1* and *lim5*) is expressed in dorsal mesoderm (*lim1* type) and ectoderm (*lim5* type) [27]. The homeobox gene *otx* is expressed in endomesoderm and anterior ectoderm in amphioxus gastrulae [25]. Expression patterns of these genes in amphioxus are quite similar to those of their orthologs in *Xenopus* (Fig. 1a) [28–35]. However, *gsc* displays a different pattern in amphioxus; its expression starts in the gastrula organizer during the early gastrula stage, and remains active throughout axial mesoderm (presumptive notochord domain) until the early neurula stage [24]. It is finally restricted to the posterior end of the notochord from early to mid-neurula stage [36] (Fig. 1a). In contrast, in vertebrates, *gsc* is expressed in the head organizer region at the late gastrula, and later in the prechordal plate, but not in the notochord [35, 37]. Based on these observations, Neidert et al. (2000) hypothesized that the shift of *gsc* expression to the anterior mesoderm during late gastrula stages is an important evolutionary event in the innovation of the head organizer, which later differentiates into the prechordal plate. However, the underlying regulatory mechanisms that enabled the change of *gsc* expression during vertebrate evolution remain largely unknown.

In *Xenopus* embryos, *gsc* is first expressed in the gastrula organizer under the influence of Wnt and Nodal signaling through the cis-regulatory module (CRM) located near the *gsc* promoter region, named *gsc*-U1 [21, 35], which includes the proximal and distal elements (DE and PE) [38–41]. The Wnt-induced homeodomain protein, Siamois and Twin, mediates activation of *gsc* [42]. Nodal signaling is transduced by phosphorylation of Smad2/3, and phospho-Smad2/3 directly upregulates *gsc* in concert with partner transcription factors FoxH1 and Wbscr11 [38, 43]. Its expression is subsequently maintained in the head organizer by Lim1 and Otx2 through the *gsc*-U1 [21, 35, 39]. In the posterior and ventral regions, *gsc* expression is repressed by the Bmp-activated, ventrally expressed, homeodomain transcription factor, Vent2 [44] and Vent1/PV.1 [39], through the same CRM, *gsc*-U1. Recently, reporter analyses using amphioxus genomic regions in mammalian cell lines and medaka fish have shown that activation of *vent* genes by Bmp and repression of *chrd* and *gsc* by Vent through their upstream regulatory regions is conserved between amphioxus and vertebrates [45, 46]. However, although these analyses have shown conserved gene regulatory networks (GRNs) for patterning along the DV axis, it remained to be determined whether GRNs in the head and trunk organizers along the AP axis are conserved. Thus, we carried out comparative analyses of CRMs for *gsc* together with those for *lim1* and *chrd*, to compare the organizers in amphioxus and vertebrates using *Xenopus* embryos. The *Xenopus* embryo is a most representative vertebrate embryo for comparison of the gastrulation process and gene expression patterns in chordates [24, 47], as both *Xenopus* and amphioxus exert the holoblastic cleavage without extraembryonic tissues. Also, the large knowledge of the organizer has been accumulated by *Xenopus* studies, as described above. Here we discuss how the chordate head evolved.

## Results

### *Bj_gsc* expression is gradually restricted to posterior mesoderm during neurulation

Before analyzing CRMs, we reexamined expression patterns of *gsc* in amphioxus using *B. japonicum* embryos (Fig. 1b–i). As shown in previous studies using *B. floridae, Bj_gsc* is expressed throughout the dorsal mesoderm in mid-gastrula (stages G5–G6, Fig. 1b–d) and late gastrula (stage G7–N0, Fig. 1e, f). In early neurula (stage N1), *Bj_gsc* expression starts to be restricted to the posterior mesoderm, but remains weak in the anterior mesoderm (Fig. 1g, h). In mid-neurula (stage N2), mesodermal expression of *Bj_gsc* is completely restricted to the posterior notochord (Fig. 1i), while *Bj_gsc* expression is detectable in anterior and posterior dorsal endoderm.

To avoid confusion, we should note that previous studies of *gsc* expression in *B. floridae* labeled embryonic stages incorrectly. First, Neidert et al. [36] described *Bf_gsc* expression localized in posterior mesoderm at mid-gastrula stage but the embryo is early neurula (stage N1). Second, Neidert et al. [36] also mislabeled mid-neurula (stage N2) with seven pairs of somite as late gastrula. Third, Yu et al. [24] described *Bf_gsc* expression in the entire dorsal mesoderm at neurula stage, but the embryo appears to be at the late gastrula to pre-hatching neurula stage (stage G7–N0). Thus, our results are consistent with previous studies and clearly describe dynamic changes of *gsc* expression during neurulation of amphioxus embryos.

### Genomic sequences of *Bj_gsc*, *Bj_lim1* and *Bj_chrd*

To compare CRMs of organizer genes between amphioxus and vertebrates, we cloned amphioxus counterparts of vertebrate CRMs for *gsc*, *lim1,* and *chrd,* from Japanese amphioxus, *Branchiostoma japonicum* (*Bj_gsc*, *Bj_lim1,* and *Bj_chrd*, respectively). Because vertebrate *gsc* is regulated through the conserved CRM, *gsc*-U1, which is located in the 0.5 kb 5′ region, we isolated the roughly 4.5 kb 5′ region, including the promoter and 5′ untranslated region (UTR) sequence of *Bj_gsc*. We also isolated the first intron of *Bj_lim1*, because *lim1* is activated by Nodal signaling in the organizer through the first intron in *Xenopus* and zebrafish [48, 49]. For *chrd*, the previous study identified a CRM near the promoter (around 1.5 kb 5′ flanking region) in Florida amphioxus, *Branchiostoma floridae* [45, 46]. However, CRMs for activation of *chrd* by Lim1 and Otx2 were not located in the promoter in *Xenopus* [21]. Therefore, we sought to identify conserved *chrd* CRMs.

**Fig. 2:**
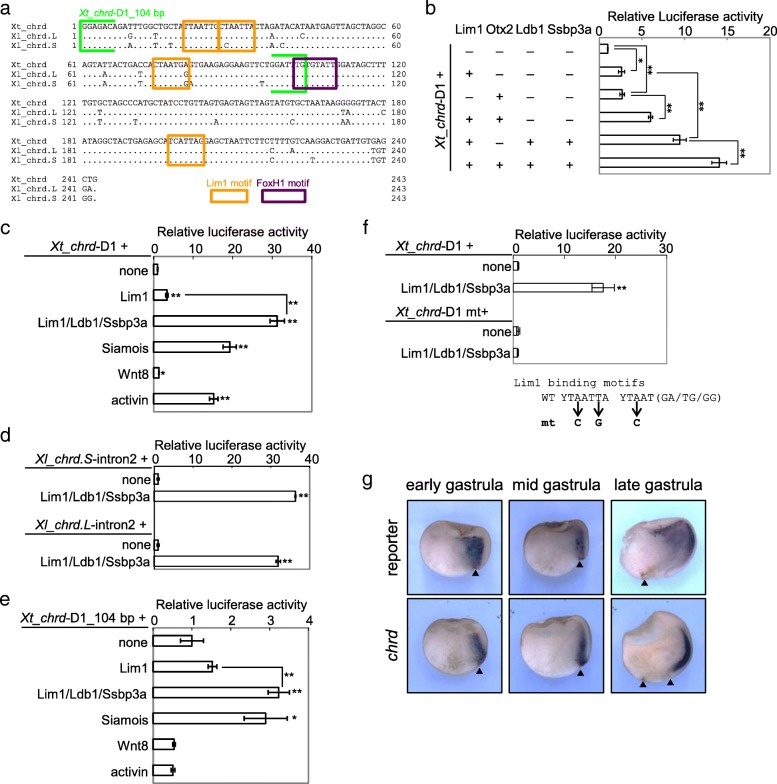
CRM activities of the second intron of *Xenopus chrd.* **a** Sequence alignment of *X. tropicalis* and *X. laevis chrd-*D1 core regions (243 bp). Orange boxes, conserved Lim1 motifs; purple box, conserved FoxH1 motif (a major partner of Smad2/3 in Nodal signaling). Siamois may bind to the Lim1 site [50]. **b**–**f** Luciferase reporter assays. *Xt_chrd*-D1 (**b**, **c**), intron 2 sequences of *Xl_chrd.L* and *.S* (**d**), *Xt_chrd*-D1_104 bp (between light green brackets in **a**) (**e**), and *Xt_chrd*-D1_mt (all four conserved Lim1 motifs are mutated) were analyzed. *Xt_chrd*-D1 showed synergistic activation by Lim1, Ldb1, Ssbp3, and Otx2 (**b**). Strong activation through *Xt_chrd*-D1 was observed in Lim1/Ldb1/Ssbp3, Siamois and activin, but not in Wnt8 (**c**). *Xl_chrd.L* and *.S* intron2 showed conserved enhancer activity, which was activated by Lim1/Ldb1/Ssbp3 (**d**). No responsiveness of *Xt_chrd* –D1_104 bp to activin (**e**) suggests that Nodal signaling activates *chrd*-D1 through the conserved FoxH1 site. Reporter activation by Lim1/Ldb1/Ssbp3 was abolished by mutating four Lim1 motifs in *Xt_chrd-D1* (**f**), indicating that Lim1 directly activates *chrd* through the intron2 enhancer. Bars represent mean ± s.e.m. *, *P* < 0.05; **, *P* < 0.01 (*t*-test, two tailed). Dosages of injected mRNAs are as follows: *lim1*, 100 pg/embryo; *ldb1*, 100 pg/embryo; *ssbp3*, 100 pg/embryo; *otx2*, 40 pg/embryo; *simois*, 100 pg/embryo; *wnt8a*, 25 pg/embryo; and *activin A*, 20 pg/embryo. **g** Transgenic reporter analysis of *Xt_chrd*-D1. Panels represent whole mount in situ hybridization of the reporter gene *mVenus* or endogenous *chrd* for transgenic embryos with dorso-ventral hemisections. Expression patterns were examined at the early (st. 10), middle (st. 11) and late (st. 12.5) gastrula stages as indicated. In total, 8 of 30 transgenic embryos showed reporter expression in the organizer. Embryos are shown with the animal pole at the top and dorsal to the right. Arrow heads, blastopore

We found enhancer activity of the second intron of *Xenopus chrd* (*Xt_chrd*, *Xl_chrd.L*, and *Xl_chrd.S*, Fig. 2a;. L and. S indicate homeologs in subgenomes of the allotetraploid frog *X. laevis* [51]), which contains a CRM bound with Lim1 and Otx2, named *chrd*-D1 [21]. Luciferase reporter assays in *Xenopus* embryos showed that Lim1, with its cofactors Ldb1 and Ssbp3a, Otx2, Siamois and Nodal signaling, activate the reporter gene through the second intron in *Xenopus* (Fig. 2b–d). The non-responsiveness of a deletion construct which lacks a conserved FoxH1 binding motif to Nodal signaling suggests that Nodal/FoxH1 signaling directly regulates *chrd* (Fig. 2e). In fact, FoxH1 binds to *chrd-D1* in *Xenopus* gastrulae [52]. In addition, a Lim1 binding motif mutated construct abolished the enhancer activity activated by Lim1/Ldb1/Ssbp3, indicating that Lim1 directly activates chrd through *chrd-D1* (Fig. 2f). Furthermore, we also performed transgenic reporter assays using *Xenopus* embryos, resulting in activation of reporter gene expression in the head organizer of gastrulae through *Xt_chrd*-D1, similar to the endogenous *chrd* (Fig. 2g). Based on these results, we cloned the second intron of *Bj_chrd* as a counterpart of vertebrate CRM for evolutionary comparisons.

**Fig. 3:**
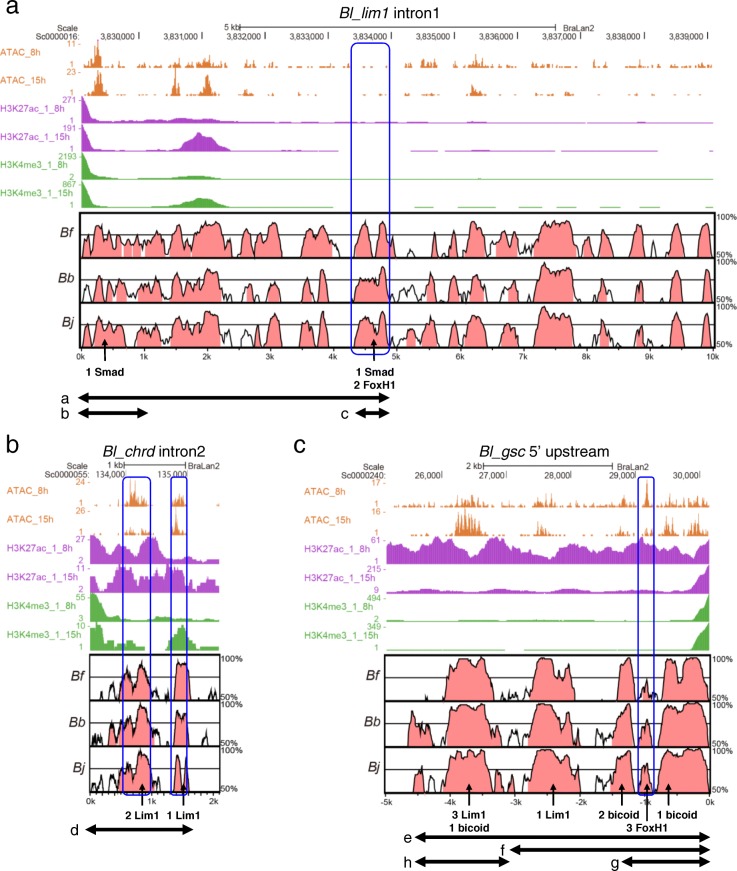
Epigenetic data from *B.lanceolatum* embryos and sequence comparisons between *B.lanceolatum, B. floridae, B. belcheri*, and *B. japonicum.* **a** ATAC-seq, H3K27ac ChIP-seq, and H3K4me3 ChIP-seq data from early gastrula (8 hpf) and early neurula (15hpf) in *Bl_lim1* intron 1 are represented with Vista plot of *Bl_lim1* intron 1 vs *Bf_lim1* intron 1, *Bb_lim1 intron1* and *Bj_lim1* intron 1. Regions with 50–100% identity were shown and conserved non-coding sequences (CNSs) were colored in red. The number of Smad motifs and FoxH1 motifs in CNSs is shown as indicated by arrows. **b** Epigenetic data in *Bl_chrd* intron 2 are represented with Vista plot of *Bl_chrd* intron 2 vs *Bf_chrd* intron 2 *Bb_chrd* intron 2 and *Bj_chrd* intron 2. The number of Lim1 sites in CNSs is indicated by arrows. **c** Epigenetic data in *Bl_chrd intron 2* are represented with Vista plot of the − 5 kb region of *Bl_gsc* vs those of *Bf_gsc, Bb_gsc* and *Bj_gsc*. The number of Lim1, bicoid, and FoxH1 sites in CNSs is shown as indicated by arrows. a–h, Regions used for reporter assays. Blue boxes indicate putative CRMs analyzed in reporter assays (Figs. 4 and 5). Additional file 1: Figure S1 shows sequence alignment of them (see Additional file 1)

Cloned genomic sequences of *Bj_gsc, Bj_lim1,* and *Bj_chrd* were then compared by Vista plot with the genome database of *Branchiostoma floridae* [53] (*Bf_gsc*, *Bf_lim1* and *Bf_chrd*, respectively), *Branchiostoma belcheri* [54] (*Bb_gsc*, *Bb_lim1* and *Bb_chrd*, respectively), and *Branchiostoma lanceolatum* [55] (*Bl_gsc*, *Bl_lim1* and *Bl_chrd*, respectively) to depict evolutionarily conserved regions as candidates of CRMs [56, 57]. There are a number of conserved regions in each pair of the sequences, in which we found conserved binding motifs for Lim1 (T/CTAATT/GA/G), Otx2 (bicoid site, TAATCC/T), FoxH1 (TGTNNATT), and Smad (GTCTG) (Fig. 3). In addition, we surveyed epigenetic data of *B. lanceolatum* embryos [55] to find more plausible CRM candidates (Fig. 3). The data show that conserved non-coding regions among *Branchiostoma* species overlap closely with open chromatin regions, which correspond to ATAC-seq peaks, in *B. lanceolatum* embryos. We next tested CRM activities of these regions using *Xenopus* embryos, as described below.

### Conservation of CRM activities of introns of *Bj_lim1* and *Bj_chrd*

**Fig. 4:**
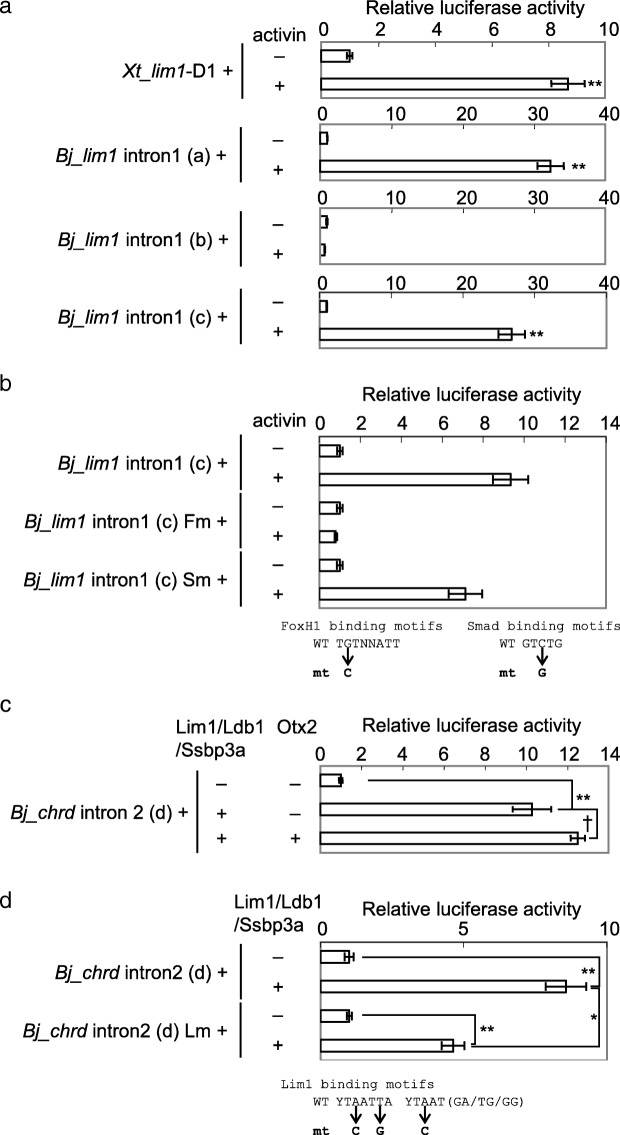
Reporter analyses of the *lim1* intron 1 and *chrd* intron 2 in the *Xenopus* embryo. **a**, **b** Luciferase reporter assays of *lim1* intron 1. Responsiveness of reporter constructs to Nodal signaling was tested with or without *activin A* mRNA (40 pg/embryo). A reporter construct mutated in two FoxH1 motifs (Fm), but not that in a Smad motif (Sm) exhibited no response to Nodal signaling (**b**), suggesting that Nodal signaling directly regulates *Bj_lim1* through FoxH1 binding to the intron1 enhancer. **c**, **d** Luciferase reporter assays of *Bj_chrd* intron 2. Responsiveness of *Bj_chrd* intron 2 (region g) to Lim1 and Otx2 was tested with or without *lim1*, *ldb1*, *ssbp3*, and *otx2* mRNAs (100, 100, 100 and 40 pg/embryo, respectively). Lim1/Ldb1/Ssbp3 significantly activated the reporter gene through *Bj_chrd* intron 2 (C), but the activation level was significantly reduced by mutating three Lim1 motifs (D). See Fig. 2f for details of the Lim1 motif mutation. Bars represent mean ± s.e.m. *, *P* < 0.05, **, *P* < 0.01; †, *P* < 0.1 (*t*-test, two tailed)

To examine the conservation of *lim1* regulation by Nodal signaling, we performed luciferase reporter assays in *Xenopus* embryos using reporter constructs, in which the previously reported activin response element (ARE) in *Xenopus lim1* intron 1 (cloned from *X. tropicalis, Xt_lim1-*D1) or *Bj_lim1* intron 1 sequences were connected upstream of the minimum promoter (Fig. 4a). The 5 kb 5′ sequence of *Bj_lim1* intron 1, but not the 1 kb sequence responds to Activin, which activates Nodal signaling (compare constructs a and b in Fig. 4a). This enhancer activity was detected in the 0.5 kb fragment at the 3′ end of the 5′ half of *Bj_lim1* intron 1 (construct c in Fig. 4a), which contains FoxH1 and Smad binding motifs. Mutation of FoxH1 binding motifs but not that of a Smad binding motif in the 0.5 kb fragment resulted in the absence of responsiveness to Nodal signaling, suggesting that *Bj*_*lim1* is induced by Nodal signaling in the gastrula organizer through the FoxH1 binding sites at the middle of its intron 1 (Fig. 4b). Thus, regulation of *lim1* by Nodal signaling in early gastrula embryos appears to be conserved in chordates.

However, those FoxH1 binding motifs are not conserved among *Branchiostoma*, although surrounding sequences are conserved (Additional file 1: Figure S1A). In addition, ATAC-seq peaks and enhancer histone marks in early embryos were not detected in the genomic region of *B. lanceolatum* corresponding to *Bj_lim1* intron 1 ARE (Fig. 3a). Although there are ATAC-seq peaks around 2 kb from 5′ end of *Bl_lim1* intron1, this region encodes a non-coding RNA gene (BL38158 gene) and enriched with promoter histone marks (H3K4me3 and H3K27ac). Therefore, some other regions may respond to Nodal signaling in *B. lanceolatum*, which should be tested in future.

To examine conservation of the organizer gene regulatory axis for *chrd*, we next performed reporter assays using reporter constructs of *Bj_chrd* intron 2 sequences. *Bj_chrd* intron 2 is activated by a combination of Lim1, Ldb1 and Ssbp3a, and mutations in Lim1 binding motifs reduced the responsiveness (Fig. 4c, d), similar to *Xt_chrd-D1* (Fig. 2b). Conservation of those Lim1 binding motifs among *Branchiostoma* species and epigenetic data of *B. lanceolatum* embryos further support the enhancer activity of *chrd* intron 2 during amphioxus development (Fig. 3b; Additional file 1: Figure S1B,C). This result suggests that regulation of *chrd* by Lim1 in early gastrula embryos is conserved in chordates. Although significant activation by Otx2 was observed in *Xt_chrd-D1* (Fig. 2b), Otx2 could not significantly activate the *Bj_chrd* intron 2 reporter with Lim1/Ldb1/Ssbp3a (Fig. 4c), implying a weaker effect of Otx on *chrd* expression in amphioxus.

### The 5′ region of *Bj_gsc* responds to Wnt and nodal, but not Lim1 and Otx

**Fig. 5:**
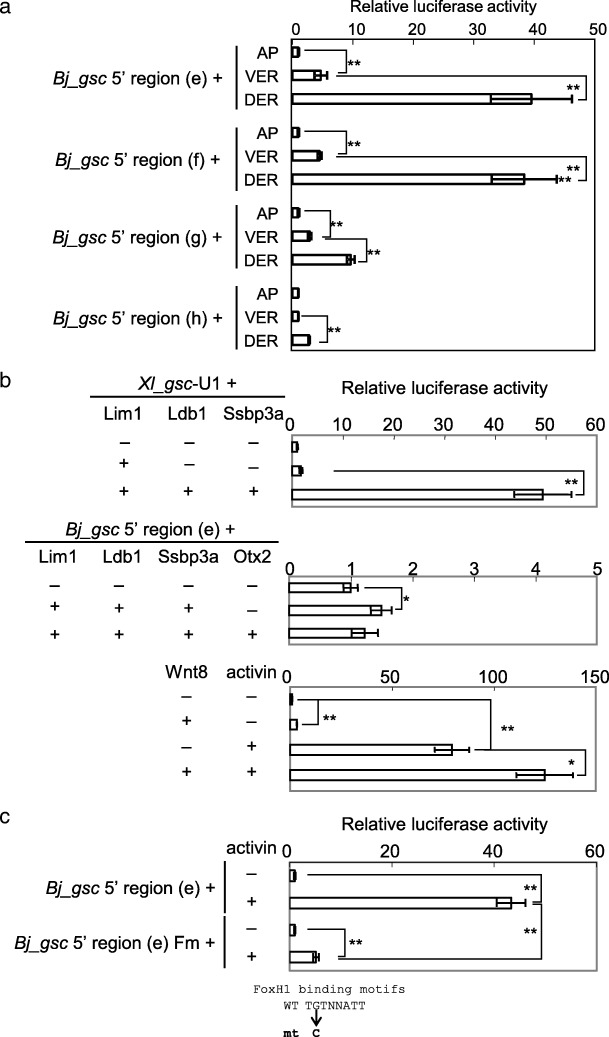
Luciferase reporter analysis of the *gsc* 5′ region in *Xenopus* embryos. **a** Luciferase reporter assays of *Bj_gsc* 5′ regions for responsiveness to endogenous factors. Reporter constructs were injected into the animal pole (AP), ventral equatorial region (VER), or dorsal equatorial region (DER) at the four-cell stage to examine responsiveness of constructs to endogenous dorsal signals. Results were normalized with activity of embryos injected with reporter constructs into the animal pole. **b** Luciferase reporter assays of *Xl_gsc*-U1 and the *Bj_gsc* 5′ region for responsiveness to exogenous factors. Lim1/Ldb1/Ssbp3a strongly activated reporter gene expression through *Xl_gsc*-U1 but only slightly through the *Bj_gsc* 5′ region. While, Wnt and Nodal signaling synergistically activated reporter gene expression through the *Bj_gsc* 5′ region. **c** Luciferase reporter assays of *Bj_gsc* 5′ region with mutations of three FoxH1 motifs for responsiveness to Nodal signaling. The mutation construct greatly reduced responsiveness to activin, suggesting that Nodal/FoxH1 signaling directly regulates *Bj_gsc* through the 5′ region. See Fig. 4b for details of the FoxH1 motif mutation. Reporter constructs were injected into the animal pole with combinations of mRNAs with dosages as follows: *lim1,* 100 pg/embryo (*Xl_gsc*-U1) or 50 pg/embryo (*Bj_gsc* 5′ regions); *ldb1,* 100 pg/embryo (*Xl_gsc*-U1) or 50 pg/embryo (*Bj_gsc* 5′ regions); *ssbp3*, 100 pg/embryo (*Xl_gsc*-U1) or 50 pg/embryo (*Bj_gsc* 5′ regions); *otx2*, 50 pg/embryo; *wnt8*, 25 pg/embryo; and *activin A*, 40 pg/embryo. Bars represent mean ± s.e.m. *, *P* < 0.05; **, *P* < 0.01 (*t*-test, two tailed)

To address how amphioxus *gsc* is regulated, we carried out luciferase and transgenic reporter analyses for *Bj_gsc* regulatory regions (Fig. 5). The data showed that the − 4.5 kb, − 3 kb, and − 1.5 kb *Bj_gsc* regions (constructs e, f, and g, respectively) are activated strongly in the marginal zone at higher levels in the dorsal side than ventral side, although the − 1.5 kb region is not sufficient for a full response. On the other hand, the − 4.5/− 3 kb region (construct h) is weakly activated only in the dorsal marginal zone but not in the ventral side. This suggests that the − 3 kb region contains CRMs responding to some factors in the dorsal mesoderm. In the − 3 kb region, there are conserved noncoding sequences overlapped with ATAC-seq peaks in *B. lanceolatum* embryos, possibly corresponding to active enhancers (Fig. 3c). We next examined what factors activate this − 3 kb region in the dorsal mesoderm.

Lim1, Ldb1, and Ssbp3 strongly activated *gsc*-U1 of *Xenopus* (upper panel of Fig. 5b), but they and Otx2 could elicit no or only slight activation of the − 4.5 kb *Bj_gsc* reporter (middle panel of Fig. 5b), in spite of the presence of several conserved motifs for Lim1 and Otx2 (Fig. 3c). By contrast, activin strongly activates the *Bj_gsc* promoter (lower panel of Fig. 5b), as has been shown in *Xl_gsc* [38, 40]. Wnt8 weakly activates the *Bj_gsc* promoter and also weakly synergizes with activin. We think that Wnt signaling indirectly activates the promoter in *Xenopus* embryos (see Discussion). Reporter constructs with mutated FoxH1 binding motifs exhibited greatly reduced responsiveness to activin, suggesting that Nodal signaling directly activates *Bj_gsc* via FoxH1 binding on conserved motifs in the 5′ region (Fig. 5c; Additional file 1: Figure S1D). These results indicate that the *Bj_gsc* promoter is activated by organizer-inducing signals Wnt and Nodal, but not by the organizer transcription factors Lim1 and Otx2, implying that the difference in expression patterns between amphioxus and vertebrate *gsc* genes (Fig. 1) is attributable to the difference in the response to Lim1 and Otx2. However, it should be noted that these data do not exclude the possible presence of Lim1/Otx-dependent CRMs that may regulate *gsc* expression in other genomic regions, and that the difference in *gsc* expression may not solely depend on the difference in CRMs analyzed here.

**Fig. 6:**
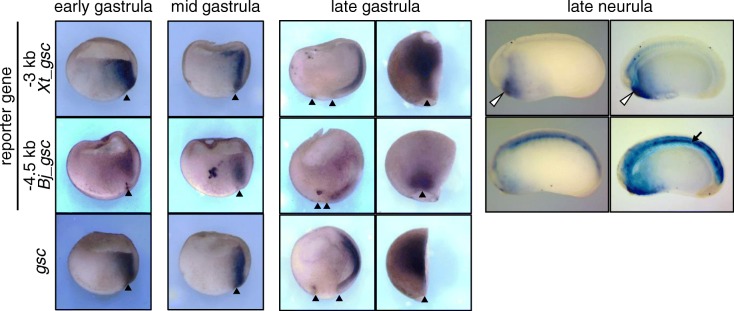
Transgenic reporter analysis of the *gsc* 5′ region in *Xenopus* embryos. Transgenic reporter assays of the − 3 kb region of *Xt_gsc* and the − 4.5 kb region of *Bj_gsc* in *Xenopus* embryos. Panels represent whole mount in situ hybridization of the reporter gene *mVenus* (first and second rows) or the endogenous *gsc* gene (third row) in dorso-ventral hemisections. Expression patterns were examined at the early (st. 10), middle (st. 11) and late (st. 12.5) gastrula stages and late-neurula stage (st. 23) as indicated. In right panels of late gastrula embryos, hemisections were represented in the dorsal view with anterior to the top. Other panels of gastrula are shown with animal to the top and dorsal to the right. Neurula embryos are shown with dorsal to the top and anterior to the left. Neurula embryos cleared in BB/BA solution are shown in right panels. Arrowheads, blastopore; open arrowheads, mouth; and arrows, notochord. Additional file 1: Figure S2 shows results in more detail (see Additional file 1)

Transgenic reporter analyses using *Xenopus* embryos further clarified the difference in dorsal expression in gastrula and neurula (Fig. 6; Additional file 1: Figure S2). In the early gastrula stage, a − 3 kb region of *Xt_gsc*, which includes CRMs-U1 to U3 [21], and a − 4.5 kb region of *Bj_gsc* both showed reporter expression in the organizer region, as well as the endogenous *gsc*, suggesting that conserved responses to Nodal signaling result in expression in the organizer region. In late gastrula stage, the − 3 kb region of *Xt_gsc* showed reporter expression in the head organizer, which recapitulates that of the endogenous *gsc*. By contrast, the − 4.5 kb region of *Bj_gsc* showed reporter expression in the posterior mesoderm, but not in the head organizer. In neurula, the − 3 kb region of *Xt_gsc* activates reporter genes around the mouth, whereas the − 4.5 kb region of *Bj_gsc* activates reporters in the notochord. In both constructs, reporter genes are frequently coactivated in the neural tissues and ventral ectoderm possibly due to the absence of CRMs for silencing in those regions (Additional file 1: Figure S2). Although the reporter expression was not restricted to the posterior notochord, expression patterns of the − 4.5 kb *Bj_gsc* reporter construct roughly resemble those of *Bj_gsc* in amphioxus gastrula to neurula (Fig. 1). The reporter expression in the anterior mesoderm derived from the organizer region disappeared in later stages possibly due to the absence of CRMs for maintaining the expression. Instead, the reporter gene was activated in the posterior mesoderm possibly through Nodal signaling because Nodal ligands are expressed in the posterior mesoderm in *Xenopus* [58–60]. Subsequently, it is maintained in the notochord of the neurula.

## Discussion

**Fig. 7:**
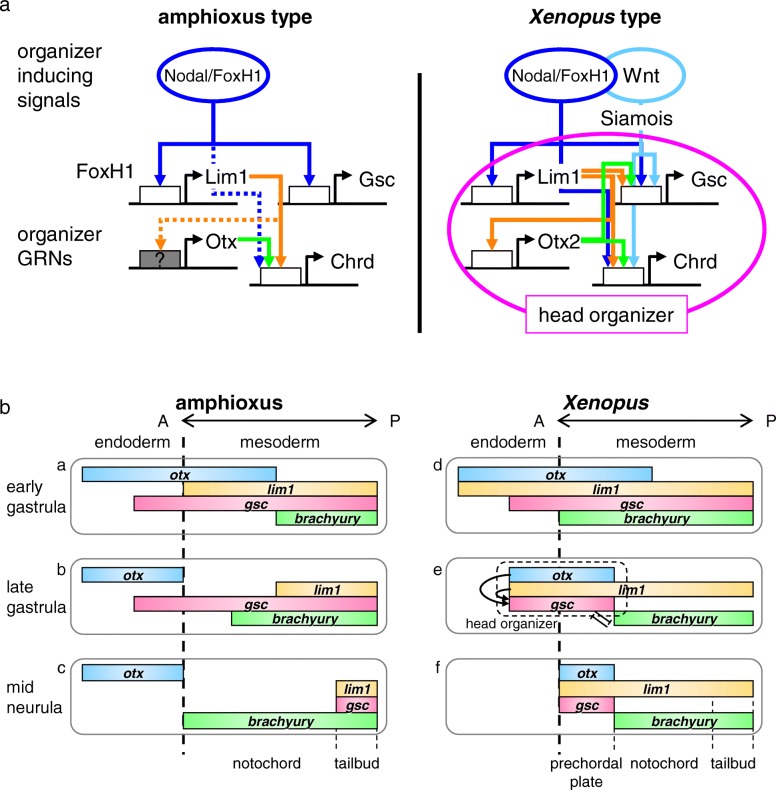
Comparisons between amphioxus and *Xenopus*. **a** Comparisons of organizer formation and organizer gene regulatory networks (GRNs) between amphioxus (left) and *Xenopus* (right). GRNs in the head organizer of *Xenopus* are shown with a magenta circle. White boxes, CRMs of each gene; gray box, CRMs of amphioxus *otx*, which have not been identified yet; dotted lines, suggested regulation [26]. **b** Comparisons of expression domains of transcription factors (*otx*, *lim1*, *gsc*, and *brachyury*) in the dorsal endoderm and the dorsal mesoderm between amphioxus (a–c) and *Xenopus* (d–f). Colored bars represent expression domains of genes at the early gastrula stage (a,d), the late gastrula stage (b,e) and the late neurula stage (c,f) with anterior to the left, as indicated. The head organizer region and regulatory interactions between transcription factors are indicated in the late gastrula stage in *Xenopus*

**Fig. 8:**
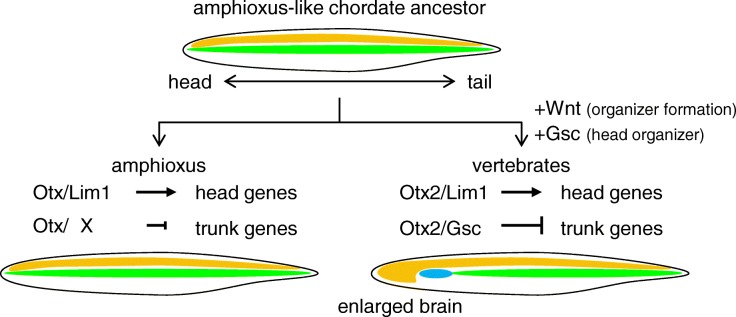
Evolutionary scenario of the vertebrate head organizer. Assuming the amphioxus-like chordate ancestor, the vertebrate ancestor should have adopted Wnt signaling for organizer formation and coopted *gsc* as a target of Lim1 and Otx2 to form the anteriorly enlarged brain by converting the anterior presumptive notochordal cells to the prechordal plate. Schematics of body plans are shown with anterior to the left and dorsal to the top. Orange, brain and neural tube; green, notochord; blue, prechordal plate

In this study, we revealed the conservation of regulation for *lim1* expression by Nodal/FoxH1 and *chrd* expression by Lim1 in the chordate gastrula organizer (Fig. 4). We also found a distinct regulatory system of *gsc* expression during gastrulation between amphioxus and vertebrates (Figs. 5 and 6). Among candidate CRMs in evolutionarily conserved sequences found by comparative genomics between *B. lanceolatum*, *B. floridae*, *B. belcheri*, and *B. japonicum* (Fig. 3), semi-conserved FoxH1 sites at the middle of *Bj_lim1* intron 1 and conserved Lim1 sites in *Bj_chrd* intron 2 are supposed to be functional (Fig. 4). However, conserved Lim1 sites and bicoid sites in the 5′ upstream region of *Bj_gsc* did not respond to Lim1 and Otx2 (Fig. 5b). These results indicate that gene cascades in the head organizer of vertebrate are only partially conserved in amphioxus (Fig. 7a). Based on these findings and the comparison of gene expression patterns (Fig. 7b), we propose an evolutionary scenario for the head organizer as described below (Fig. 8).

In this scenario, Nodal is the ancestral organizer-inducing signal, upregulating *lim1*, *gsc*, and *chrd* (Figs. 4 and 5, and [26]). By contrast, Wnt signal is thought to have been co-opted for organizer formation in ancient vertebrates to activate an additional organizer gene, like the homeobox gene *siamois* in *Xenopus* or *bozozok* in zebrafish [12, 15], because in amphioxus no dorsal accumulation of nuclear-catenin is observed in blastula to gastrula embryos and LiCl neither dorsalizes the embryo [61] nor activates *gsc* expression [62]. However, unexpectedly *Bj*_*gsc* was activated by Wnt8 to some extent in the reporter assay using *Xenopus* embryos (Fig. 5b). There are two possibilities to be tested: (i) in *Xenopus* embryos, Wnt signaling induces *chrd* and Chrd relieves the *Bj*_*gsc* reporter from repression by Bmp signaling through Vent2 [45], or (ii) Wnt signaling enhances Nodal signaling through stabilizing Smad3 [63, 64]. Together with our results, it is likely that the anterior expression domain of *gsc* was acquired in the vertebrate lineage by co-option or creation of the CRM, which responds to Lim1 and Otx2. This CRM evolution may have been triggered by two rounds of whole-genome duplication before the emergence of vertebrates, which could have released them from evolutionary and developmental constraints of genes.

What is the molecular consequence of *gsc* cooption in the head organizer? A plausible answer comes from our recent study on the basis of genome-wide analysis using *Xenopus* embryos [21]. That study suggests that Otx2 upregulates head organizer genes, such as *gsc, cerberus*, and *chrd*, in cooperation with Lim1 through enhancers named “type I CRMs,” whereas Otx2 downregulates trunk organizer genes, such as *brachyury*, *wnt8a* and *not*, with Gsc through silencers named “type II CRMs” [21]. The paper proposed that the bilaterian-conserved head selector, Otx, marks genes to be regulated in the head, and that its partner transcription factors that activate or repress Otx-marked genes can differ between organisms to make diverse head structures [21]. That is, the repression system of trunk organizer (notochord) genes in the anterior region of dorsal mesoderm may have been established by coopting *gsc* as a target gene for Lim1/Otx and by integrating Gsc as a repressive partner of the head selector, Otx. This possibility is also supported by previous reports. First, *otx* is expressed in the head region of all bilaterians and has a fundamental role to repress trunk genes such as *hox* genes [65, 66]. Second, Gsc can bind to the same monomer binding motif as Otx, the bicoid site (TAATCY) [21, 39, 50, 67, 68], and has been predicted to form a heterodimer complex with Otx on the P3C site (TAATCNNATTA) [21, 69]. Third, even though *gsc* and *brachyury* in amphioxus are coexpressed in the dorsal mesoderm [25, 36, 70, 71], Gsc does not appear to repress *brachyury*, in contrast to vertebrate *brachyury*, which is repressed by Gsc and Otx2 [17, 18] (Fig. 7b). Our results also suggest that CRMs of *brachyury* in the vertebrate ancestor evolved to respond to a complex of Gsc and Otx for downregulation.

Incorporating our experimental data, we revisit the comparison of gene expression patterns between amphioxus and *Xenopus* (Fig. 7b), which is composed of previously reported expression data [24, 25, 27, 28, 30–32, 34, 36, 37, 47, 70–72]. In amphioxus, *otx*, *lim1*, and, *gsc* are all coexpressed in anterior mesoderm in the early gastrula stage (panel a in Fig. 7b), but possibly due to the absence of their regulatory interactions, *otx*, *lim1*, and *gsc* are turned off in the anterior part until the mid-neurula stage (panel b,c). *brachyury* expression is overlapped with *lim1* and *gsc* in the posterior dorsal mesoderm in the early gastrula stage, and extends to the anterior during gastrulation. Then, the *brachyury*-expressing region develops into the notochord, and the posterior tip develops into the tail bud where *lim1*, *gsc*, and *brachyury* are expressed (panel c). In *Xenopus*, regulatory interactions between *otx2*, *lim1*, *gsc*, and *brachyury* have been established, thereby maintaining *otx2*, *lim1*, and *gsc* expression and repressing *brachyury* in the anterior part of mesoderm to form the head organizer (panels d and e). As a consequence, the anterior part develops into the prechordal plate, whereas the posterior part develops into the notochord (panel f). Thus, in the vertebrate ancestor, three major events are thought to have occurred. (i) *lim1* and *otx* acquired type I CRMs (by mutations or cooption) to maintain their own expression through an auto-regulatory loop; (ii) *gsc* acquired type I CRMs to be upregulated by Lim1 and Otx2; and (iii) *brachyury*, as well as other posterior genes, acquired type II CRMs to be downregulated by Gsc and Otx2. The key event was to have created a new GRN for repression of trunk organizer genes by Gsc and Otx, which are maintained by Lim1 and Otx in the anterior mesoderm. This GRN converts the notochordal mesoderm (trunk organizer) to the head organizer and later prechordal plate, leading to acquisition of the vertebrate-type head (Fig. 8). This hypothetical model should be addressed in the future by functional analyses of those transcription factors and reporter assays for potential CRMs in amphioxus embryos as performed previously [25, 26, 46, 73, 74].

Another aspect in the evolution of the vertebrate head mesoderm has also been examined experimentally by comparing vertebrates and amphioxus [47]. Onai et al. showed that mesodermal involution is a key developmental event to segregate dorsal mesoderm anteroposteriorly. In amphioxus, gastrulation occurs by simple invagination with little mesodermal involution, in contrast to vertebrate gastrulation which takes place through massive cell movements such as involution and convergent extension. Interestingly, inhibition of mesodermal involution in *Xenopus* embryos recapitulated amphioxus-like dorsal mesoderm formation, in which segregation of head-trunk organizers did not occur. Taken together with this study, both GRNs and cell movements during gastrulation must have been drastically rearranged in the vertebrate lineage to evolve the head.

How have the gastrula organizer and head organizer evolved among eumetazoans? Evolutionary data for the gastrula organizer has accumulated through studies of chordate outgroups, such as the sea anemone, *Nematostella* (reviewed in [75]). It has been reported that the blastopore lip of *Nematostella* has secondary axis-inducing activity when transplanted into *Nematostella* embryos [76], and that many organizer-related genes including *lim1*, *otx*, *foxa*, and *brachyury*, but not *gsc*, are expressed around the blastopore [77–81]. Although a report with *Nematostella* showed that *gsc* is expressed in early gastrula endoderm [82], the detected expression was very weak and leaky compared to other ancestral organizer genes. These reports suggest that *gsc* was not an ancient organizer gene and that it was recruited to the organizer at least after bilaterians arose. In both polychaetes (Protostomes) and sea urchins (Deuterostomes), *gsc* and *brachyury* are coexpressed in the stomodaeum region (oral region) [83, 84], which is similar to the situation in the dorsal mesoderm of amphioxus. It was reported that knockdown of *gsc* in sea urchin embryos eliminates *brachyury* expression in the oral ectoderm [85], which contrasts with the situation in vertebrates, suggesting that the deuterostome ancestor did not have the negative regulatory axis from Gsc to *brachyury*. On the other hand, no evidence was found for positive regulation of *gsc* by Lim1 and Otx in any eumetazoans, except for vertebrates. Thus, it is probable that the positive regulatory axis from Lim1 and Otx to *gsc* and the negative regulatory axis from Gsc to *brachyury* is unique to vertebrates.

Neidert et al. [36] raised the question whether the ancestral chordate had (i) the amphioxus-type of head with the notochord and no anterior *gsc* expression or (ii) the vertebrate-type of head with the prechordal plate and anterior *gsc* expression. On the basis of our data and previous reports, we prefer the model (i) that the head organizer as well as the prechordal plate evolved in the vertebrate ancestor, which was initiated by cooption of *gsc* as a Lim1/Otx target gene (Fig. 8).

## Conclusions

Gene regulatory networks conserved in chordates, and those specific to vertebrates, illustrate that rearrangement of CRMs for *gsc* is a key event for the evolution of the vertebrate head organizer. Co-option of *gsc* into the anterior mesoderm lead to further rearrangements of GRNs for head formation, resulting in the evolution of the vertebrate head with anteriorly enlarged brain.

## Materials and methods

### Animals

Adult male and female *Xenopus laevis* were purchased from Sato Breeder and Xenopus Yoshoku Kyozai (Japan), and maintained in our frog facility. Mature adult amphioxus, *Branchiostoma japonicum*, were collected in the coastal waters of the Enshu Nada Sea, Japan, during the breeding season.

### Whole-mount in situ hybridization of amphioxus embryos

The coding sequence of *Bj_gsc* was cloned into pGEM-T vector (Promega) by conjugating three exon sequences PCR-amplified from genomic DNA of *B. japonicum* using In-Fusion HD Cloning kit (Takara). Whole-mount in situ hybridization of amphioxus embryos was performed as described [86], using digoxigenin-labelled anti-sense probes for *Bj_gsc*, which was transcribed from linearized plasmids.

### Microinjection experiments in *Xenopus* embryos

*X. laevis* fertilized eggs were dejellied and injected with mRNAs and reporter DNA. For mRNA synthesis, coding sequences of *Xenopus* genes used in this study were cloned into the pCSf107mT vector, which contains the SP6/T7 terminator [87]. Capped mRNA was synthesized using the mMESSAGE mMACHINE SP6 kit (Ambion). For luciferase assays, reporter plasmid DNA (50 pg/embryo) with mRNAs for *lim1*, *ldb1*, *ssbp3*, *otx2*, *siamois*, *wnt8a*, or *activin A* (dosages are indicated in figure legends) were injected together into the animal pole region of both blastomeres at the two-cell stage.

### Cloning of cis-regulatory regions

The 5′ region of *Bj_gsc*, *Bj_lim1* intron 1 and *Bj_chrd* intron 2 were PCR-cloned with primers designed from the genome sequence of *B. floridae* (Joint Genome Institute, assembly version 1.0). Using sequences of obtained partial genomic DNA fragments, inverse PCR was performed to determine genomic sequences around the primers. Genomic sequences of *B. japonicum* have been deposited in the DDBJ as follows: 5′ upstream region of *Bj_gsc* (AB972409), the first intron of *Bj_lim1* (AB972410), and the second intron of *Bj_chrd* (AB972411). Second introns of *Xt_chrd, Xl_chrd.L* and *Xl_chrd.S* were PCR-cloned with primers designed from the second and third exons using genomic DNA of N8A2 strain of *X. tropicalis* (the same strain as that used for the genome sequence in [88]) and J-strain of *X. laevis* (the same strain as that used for the genome sequence in [51]).

### Epigenetic data and vista plot

ATAC-seq data and ChIP-seq data (H3K4me3, H3K27ac, and H3K27me3) of *B. lanceolatum* embryos [55] were visualized with UCSC genome browser track hubs (https://genome-asia.ucsc.edu/cgi-bin/hgTracks?db=hub_78274_BraLan2). Vista plots were generated with Vista tool (http://genome.lbl.gov/vista/index.shtml) using the *B. lasceolatum* genomic sequence as the X-axis to combine with the epigenetic data. The following sequences were used for consensus binding motifs of transcription factors; (C/T)TAAT(T/G)(A/G) for Lim1, TAATC(C/T) for Otx2 (bicoid sites), TGTNNATT for FoxH1, and GTCTG for Smad [21, 39, 49, 50, 67, 68, 89–91].

### Luciferase assay using the *Xenopus* embryo

Luciferase assays were performed as described [92]. To make reporter constructs for *Bj_gsc* (e, f, g in Figs. 3 and 5), 5′ regions upstream from the start codon of *Bj_gsc*, which include the promoter, were connected to the start codon of the *luciferase* reporter gene in the pGL3 vector (Promega). Other constructs were made by inserting a genomic fragment into the pGL4.23 vector (Promega), which has an artificial, minimal promoter. Luciferase activity was analyzed at the gastrula stage (st 10.5–11). The mean and standard error were calculated by assaying five pools of three embryos for each injection sample. The results were normalized with activity of embryos injected with reporter constructs alone, and displayed as relative activity. Statistical significance was examined with Student′s or Welch′s *t*-test after *F*-test.

### Transgenic analysis in the *Xenopus* embryo

Transgenesis was performed with sperm nuclear transplantation into unfertilized eggs with restriction-enzyme-mediated integration (REMI) methods as described [93–95]. Reporter constructs were made using a pGL4.23 derived vector, pGL4.23 mV, in which the luciferase gene was replaced with the coding sequence for the monomeric Venus fluorescent protein (mVenus, made by Y. Mii, Y. Honda and M. T.). A genomic fragment was inserted into the pGL4.23 mV vector as described for luciferase reporter constructs. Embryos were fixed at appropriate stages with MEMFA for 2 h, dehydrated with ethanol and stored at − 20 °C overnight. Fixed embryos were mounted in a drop of 2% agarose and 0.3 M sucrose in 1 x PBS on a plastic dish [21, 35]. Agarose-mounted embryos were bisected along the dorso-ventral axis, in which one half was used for detection of the reporter gene and the other half was used for detection of endogenous mRNAs. Hemisections were refixed with MEMFA for 1 h and dehydrated with methanol before whole mount in situ hybridization (WISH). WISH was performed as described [96] using digoxigenin-labelled anti-sense probes for *mVenus*, *Xl_gsc*, and *Xl_chrd*, which was transcribed from linearized plasmids. After treatment with alkaline phosphatase-conjugated anti-DIG antibody (Roche; 1/2000 diluted), chromogenic reaction was performed with BM Purple (Roche). Stained embryos were refixed with Bouin solution (saturated picric acid solution: 37% formaldehyde solution: acetic acid = 15: 5: 1), bleached with peroxide, and cleared in BB/BA solution (benzyl benzoate: benzyl alcohol = 2:1) for observation.

## Additional file

Additional file 1 **Figure S1.** Sequence alignment of CNSs examined in reporter assays **a** Alignment of a CNS in the middle of *lim1* intron 1 of *Branchiostoma* species (analyzed in Fig. 4b). A Smad motif is completely conserved among *Branchiostoma*, but two FoxH1 motifs in *Bj_lim1* are not conserved in other genomes. **b** Alignment of a CNS in the 5′ half of *chrd* intron 2 of *Branchiostoma* species (analyzed in Fig. 4d). In addition to two conserved Lim1 motifs, two FoxH1 motifs are also conserved among *Branchiostoma*. **c** Alignment of a CNS in the 3′ half of *chrd* intron 2 of *Branchiostoma* species (analyzed in Fig. 4d). In addition to a conserved Lim1 motif, a FoxH1 motif is also conserved among *Branchiostoma*. **d** Alignment of a CNS in the − 1 kb region of *gsc* of *Branchiostoma* species. Three FoxH1 motifs are conserved among *Branchiostoma*. **Figure S2.** Details of transgenic reporter analysis of the *gsc* 5′ region in *Xenopus* embryos. Panels represent representative embryos in transgenic reporter assays. In early neurula, reporter gene of the − 0.5 kb *Xt_gsc* construct is likely to be expressed in the prechordal plate, whereas − 4.5 kb *Bj_gsc* constcuts showed reporter expression in mesoderm (notochord) and ectoderm (neural tube) of the dorsal midline. In late neurula, − 3 kb *Xt_gsc* constructs often showed reporter expression in the neural tissue, but never in the notochord. Panels of cleared embryos are indicated with BB/BA at the right bottom. Embryos are shown in the same orientation as in Fig. 6, except for the right panel of early neurula expressing the − 4.5 kb *Bj_gsc* reporter, which is shown in the dorsal view with anterior to the left. Arrowheads, blastopore; open arrowheads, mouth; and arrows, notochord. The bottom table represents a summary of transgenic reporter assays (“expr.” means expression). (PDF 188 kb)

## Data Availability

The datasets supporting the conclusions of this article are included within the article.
