# Supplementary material for: Evolution of cis-regulatory modules for the head organizer gene goosecoid in chordates: comparisons between Branchiostoma and Xenopus
Source: Zoological Lett. 2019 Aug 2;5:27. doi: 10.1186/s40851-019-0143-1 (PMC6679436; doi:10.1186/s40851-019-0143-1)
Supplement: Supplementary file 1 — Figure S1. Sequence alignment of CNSs examined in reporter assays a Alignment of a CNS in the middle of lim1 intron 1 of Branchiostoma species (analyzed in Fig. 4b). A Smad motif is completely conserved among Branchiostoma, but two FoxH1 motifs in Bj_lim1 are not conserved in other genomes. b Alignment of a CNS in the 5′ half of chrd intron 2 of Branchiostoma species (analyzed in Fig. 4d). In addition to two conserved Lim1 motifs, two FoxH1 motifs are also conserved among Branchiostoma. c Alignment of a CNS in the 3′ half of chrd intron 2 of Branchiostoma species (analyzed in Fig. 4d). In addition to a conserved Lim1 motif, a FoxH1 motif is also conserved among Branchiostoma. d Alignment of a CNS in the − 1 kb region of gsc of Branchiostoma species. Three FoxH1 motifs are conserved among Branchiostoma. Figure S2. Details of transgenic reporter analysis of the gsc 5′ region in Xenopus embryos. Panels represent representative embryos in transgenic reporter assays. In early neurula, reporter gene of the − 0.5 kb Xt_gsc construct is likely to be expressed in the prechordal plate, whereas − 4.5 kb Bj_gsc constcuts showed reporter expression in mesoderm (notochord) and ectoderm (neural tube) of the dorsal midline. In late neurula, − 3 kb Xt_gsc constructs often showed reporter expression in the neural tissue, but never in the notochord. Panels of cleared embryos are indicated with BB/BA at the right bottom. Embryos are shown in the same orientation as in Fig. 6, except for the right panel of early neurula expressing the − 4.5 kb Bj_gsc reporter, which is shown in the dorsal view with anterior to the left. Arrowheads, blastopore; open arrowheads, mouth; and arrows, notochord. The bottom table represents a summary of transgenic reporter assays (“expr.” means expression). (PDF 188 kb) [file 40851_2019_143_MOESM1_ESM.pdf]

Figure S1

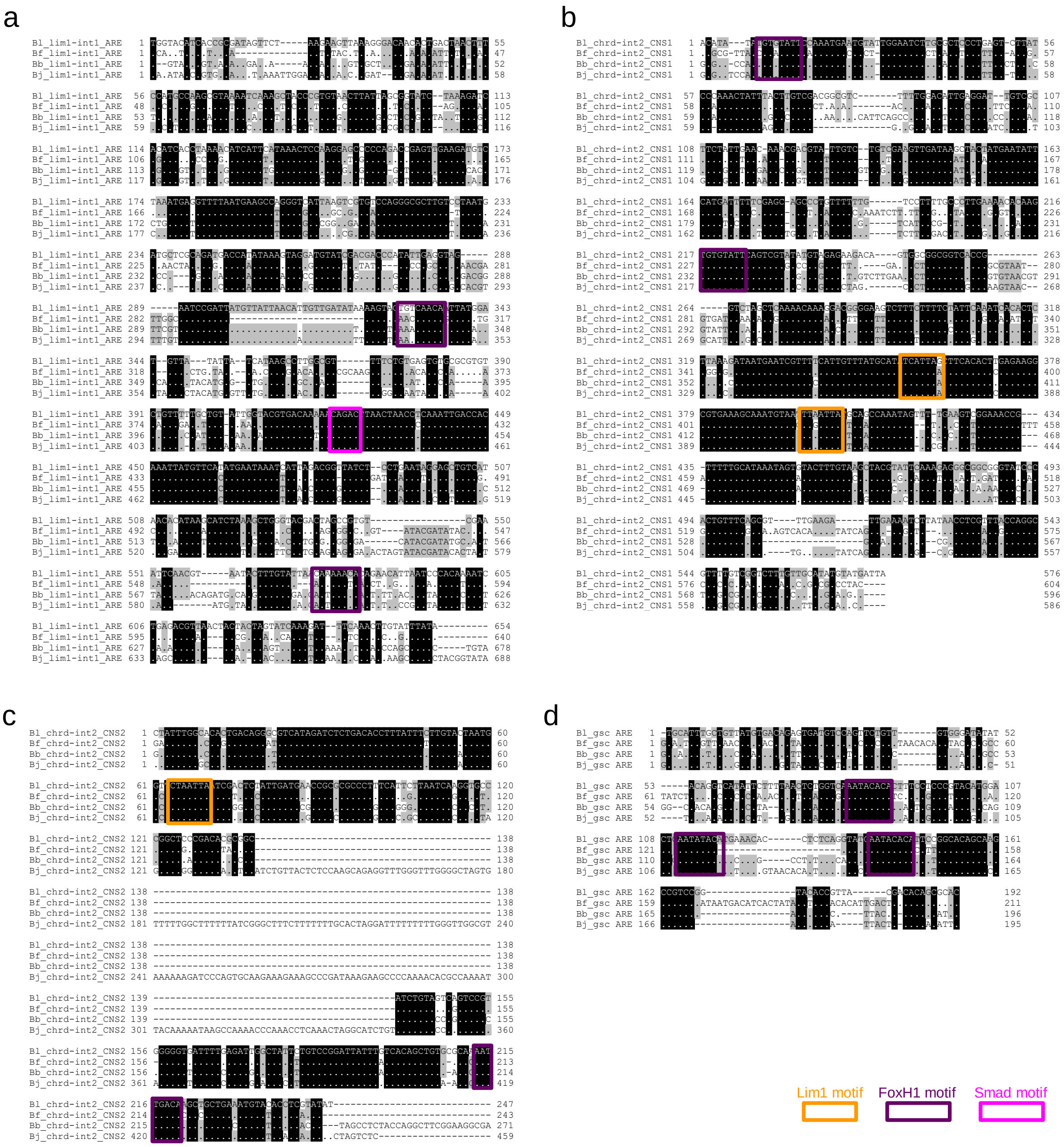

Figure S2

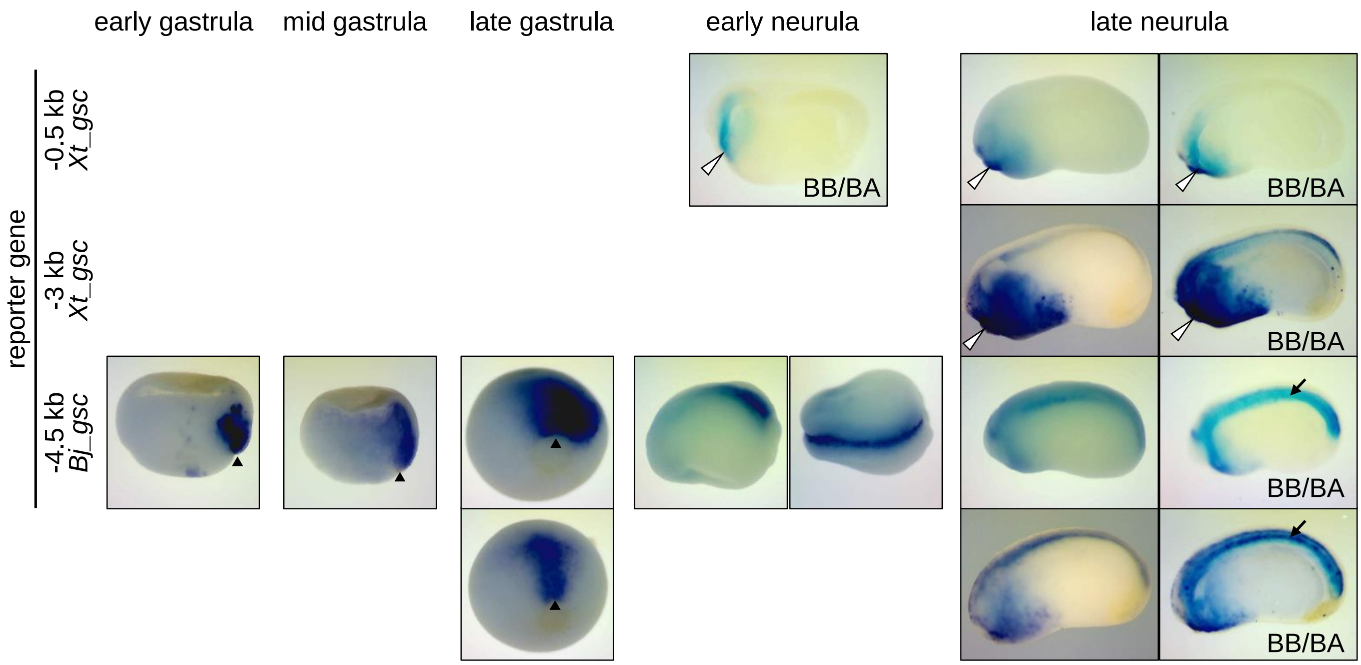

Reporter	Stage	Reproducible expr.	Ectopic expr.	No expr.	Total
-0.5 kb *Xt_gsc*	Mid-Late Neurula	13 (Mouth)	0	9	22
-3 kb *Xt_gsc*	Early-Mid gastrula	13 (Organizer)	6	22	41
	Late gastrula	14 (Head organizer)	9	33	56
	Mid-Late Neurula	13 (Mouth + neural tissue)	1	2	16
-4.5 kb *Bj_gsc*	Early-mid gastrula*	5 (Organizer)	1	17	23
		28 (Organizer)	14	6	48
	Late gastrula*	2 (Posterior mesoderm)	3	26	31
		17 (Posterior mesoderm)	3	10	30
	Mid-Late Neurula*	19 (notochord + neural tissue)	2	13	34
		40 (notochord + neural tissue)	12	22	74

*results of two independent experiments are shown in separate lines
